# Healthcare-Associated Ventriculitis in Children during COVID-19 Pandemic: Clinical Characteristics and Outcome of a First Infection

**DOI:** 10.3390/antibiotics12101501

**Published:** 2023-09-30

**Authors:** Jesús David Licona-Enríquez, María Guadalupe Labra-Zamora, Alma Griselda Ramírez-Reyes, María Guadalupe Miranda-Novales

**Affiliations:** 1 Infectious Diseases Department, Pediatric Hospital, National Medical Center, XXI Century, Mexican Institute of Social Security, Mexico City 06720, Mexico; jdliconae@gmail.com (J.D.L.-E.); dra_labra@hotmail.com (M.G.L.-Z.); 2 Neurosurgery Department, Pediatric Hospital, National Medical Center, XXI Century, Mexican Institute of Social Security, Mexico City 06720, Mexico; alma.ramirezr@imss.gob.mx; 3 Analysis and Synthesis of Evidence Research Unit, National Medical Center, XXI Century, Mexican Institute of Social Security, Mexico City 06720, Mexico

**Keywords:** COVID-19 pandemic, CSF (cerebrospinal fluid) diversion procedures, healthcare-associated ventriculitis, neurosurgery, surgical site infection

## Abstract

During the COVID-19 pandemic, patients in need of neurosurgical care suffered. Elective procedures were postponed, and emergency care visits decreased. Healthcare-associated ventriculitis (HAV) is a serious problem in children, with poor outcomes and frequent relapses. Our objective was to describe the clinical characteristics and the factors associated with a first HAV in children during two years of the pandemic. A retrospective cross-sectional study was performed from January 2021 to December 2022. The inclusion criteria were patients who developed a first HAV after a primary cerebrospinal fluid diversion procedure. The controls included patients without a first infection. Intraoperative and clinical data were extracted from medical records. A total of 199 CSF diversion surgeries were registered. A first infection occurred in 17 patients (8.5%), including 10 with external ventricular drain (EVD) and 6 with ventricular shunts. Gram-positive cocci were identified in 70.6%. Six patients recovered uneventfully, eight had relapse or superinfections, and three eventually died. Twenty patients were included as controls. Factors associated with a first infection were a younger age (median 9 vs. 102 months, *p* < 0.01), malnutrition (23.5% vs. 0%, *p* = 0.03), and an EVD placement (58.8% vs. 10%, *p* = 0.03). None of the intraoperative factors showed statistically significant differences. The rate of HAV was high. Most cases presented in children <1 year and with an EVD.

## 1. Introduction

During the COVID-19 pandemic, medical and surgical care adjusted to the health emergency. Routine and elective procedures were postponed. At the beginning, people avoided visits to the emergency rooms due to the fear of contagion [1,2]. Few neurosurgery procedures were deferred, as progression and deterioration of clinical condition are expected in patients with longer waiting times. Cerebrospinal diversion surgeries are the most common procedures in the Pediatric Hospital, National Medical Center, a tertiary-care-level reference institution. As with any surgical procedure, the placement of a cerebrospinal fluid (CSF) shunt system involves risks of complications; the most common are obstruction, infection, pseudocyst, bowel perforation, and subdural hematoma. Infections, whether localized or systemic, correlate with medium- and long-term impaired mental capacity [3,4].

The surveillance of ventriculitis associated with CSF shunt systems or external ventricular drains (EVD) is difficult. There is a lack of a standard definition. The Centers for Disease Control and Prevention’s National Healthcare Safety Network (CDC/NHSN) include definitions for post-operative (surgical site) infection, but not for shunt infections specifically [5]. The Hydrocephalus Clinical Research Network (HCRN) proposed a consensus definition: (a) microbiological determination of bacteria present in a culture or Gram stain of CSF, wound swab, and/or pseudocyst fluid; (b) shunt erosion (visible hardware); (c) abdominal pseudocyst (without positive culture); or, for children with ventriculo-atrial shunts, (d) presence of bacteria in a blood culture [6]. The HCRN definition has not been widely adopted, and comparison among centers is difficult. Moreover, there are no definitions involving ventriculostomies or EVD [7]. The published rate of infections varies greatly (0–45%), and children are at higher risk of infection in comparison to adults [4,7,8,9,10].

CSF shunt systems can become infected by colonization during the surgery due to an ascending infection from the distal end of the shunt (for example, bowel perforation), or by bloodstream dissemination. After contamination or colonization, biofilm formation plays an important role in the development of the infection and avoids the elimination of the microorganisms with antibiotics [11]. EVD often colonizes after the first seven days of insertion. Frequent manipulation for sampling, non-adherence to insertion and maintenance protocols, and longer duration are associated with higher rates of infection. There is no evidence to support the catheter exchange at a defined interval to reduce the infection rate [12]. In middle- and low-income countries, causative bacteria are mostly Gram-positive cocci (*Staphylococcus epidermidis* and other coagulase-negative species, *Staphylococcus aureus,* and *Enterococcus* spp.), followed by enteric and non-fermenting Gram-negative bacilli *(Escherichia coli*, *Klebsiella pneumoniae*, *Enterobacter* spp., *Pseudomonas aeruginosa*, and *Acinetobacter baumannii*) [9,10,13,14,15].

More than 50% of infections take place in the first month following the diversion procedure. In children, several risk factors have been associated with the development of infection: timing and type of surgical prophylaxis, the surgeon’s experience, timing and duration of the surgery, type/etiology of hydrocephalus, younger age, repeated shunt revisions, and lack of a surgical protocol to minimize shunt-associated infections, among others [16,17].

Symptoms of infection may include fever, headache, nausea, vomiting, lethargy, irritability, and changes in mental status, but in many cases, clinical presentation is vague and diagnosis may be delayed [6,7,8].

During the pandemic, the Pediatric Hospital was one of the three pediatric hospitals in Mexico City designated to provide care for COVID-19 patients, and was converted to a hybrid hospital to continue providing attention to specialty patients, as well as children with moderate to severe SARS-CoV-2 infection.

The objective of this study is to describe the clinical characteristics and the factors associated with a first infection in children after a primary ventricular drain procedure during two years of the COVID-19 pandemic (2021–2022).

## 2. Results

### 2.1. Clinical Presentation

During the two-year study period, a total of 199 ventricular diversion procedures were performed in the pediatric hospital: 117 EVD, 81 VPS, and 1 ventriculo-atrial shunt. The procedures included ventriculo-peritoneal shunt (VPS) placements and revisions, ventriculostomies, changes from ventriculostomy to VPS or from VPS to ventriculostomy, and ventriculostomy after removal of a VPS due to infection.

In comparison with the pre-pandemic period (2017–2019), the rate of HAV increased starting from the beginning of the COVID-19 pandemic (2020). The highest rate (25.5%) was registered during 2021 (Table 1). The study period (2021–2022) was selected because the Pediatric Hospital returned to providing full routine care by the end of 2020. During the pre-pandemic period, the microorganisms that caused HAV had a predominance of Gram-positive cocci (55–61.4%), followed by enterobacterales (24.2–33%). 

Of the 199 CSF diversion procedures in 2021–2022, a first HAV occurred in 17 patients (8.5%). Infection was more common in males younger than one year (median age: 9 months). The most frequent indication for the procedure was a congenital central nervous system (CNS) malformation. Two were preterm infants. More than half of the patients had an EVD, and seven had a ventricular shunt (six with VPS and one a ventriculo-atrial shunt). The antibiotic used for prophylaxis was adequate for most of the procedures according to the hospital guidelines (cephalothin), but it was seldom administered on time. Common practices were prescriptions after the end of the procedure, or in the subsequent 24 h. There was no justification in the medical charts for the prescription of a different antibiotic in five patients. The median duration of the procedures was one hour (Table 2).

Eleven of the seventeen patients presented with signs or symptoms that led to the suspicion of infection. The most common sign was fever. In the rest, infection was suspected due to the macroscopic changes in the CSF, as all of them had an EVD. Infection was confirmed by a positive CSF culture in all patients. Blood cultures were taken in patients with fever, and all were negative. None of the patients had signs of severe sepsis. Gram-positive cocci were the predominant bacteria (70.5%). Only six patients recovered uneventfully with the first course of antibiotics, and a VPS was placed after completing the antibiotic treatment. Four patients had a relapse of the initial infection with the same microorganism, and four had superinfections with a different microorganism (Table 2).

The laboratory analysis of the CSF showed high median WBC (white blood cell) counts and an increased amount of proteins and lactate, but the median glucose value was normal. The minimum and maximum values varied greatly (Table 3); two patients had normal parameters in their CSF samples. Parameters in the blood also showed a broad range of values. Despite the fact that only 11 patients had at least one sign of infection, the median white blood cell count/mm^3^, median value of total neutrophils, and median value of C-reactive protein were above normal levels. Serum glucose levels were not taken at the same time as the CSF samples; therefore, comparisons could not be made.

### 2.2. Time of Infection and Management

HAV presented seven days (median) after the procedure in patients with EVD (interquartile range IQR 3–8 days), but varied considerably for VPS; three cases presented in the first month (at 5 days, 16 days, and 17 days), while the other three occurred 10, 11 and 12 months after the surgery. In the patient with the ventriculo-atrial shunt, infection presented 15 days after the operation. Once the positive CSF culture has been informed, all ventricular catheters and shunt systems were removed, an EVD was placed, and proper antibiotic therapy was initiated according to the susceptibility pattern.

Antibiotic treatment for patients with Gram-positive cocci consisted of intravenous vancomycin plus oral rifampicin in seven cases, intravenous vancomycin in three, and intravenous linezolid in two. The choice of antibiotic treatment depended on the attending physician. All coagulase-negative staphylococci and one *Staphylococcus aureus* were methicillin-resistant. The median time to the first negative culture was five days (minimum 2 d and maximum 7 d). The duration of antibiotic treatment varied from 7 to 21 days; 4 of 12 patients were cured with the first course of antibiotics.

For the five patients with Gram-negative bacilli infections, the standard treatment included meropenem, and all isolates showed in vitro susceptibility to carbapenems. *E. coli* isolates were extended spectrum beta-lactamase (ESBL) producers. Only two patients with *E. coli* were cured with the first course of antibiotics. One patient with *E. coli* infection also received intraventricular amikacin due to the persistence of positive CSF cultures, and one patient with *E. cloacae* also received intravenous ciprofloxacin. The total antimicrobial treatment varied from 14 to 26 days. 

#### Relapse and Superinfections

Three patients with Gram-positive cocci infections and one with *E. cloacae* had relapses and required extended courses of antibiotics and second EVD exchanges. Four patients with coagulase-negative cocci infections developed superinfections or second infections with different microorganisms, such as *Stenotrophomonas maltophilia*, *Klebsiella pneumoniae*, *Staphylococcus hominis*, and *Staphylococcus lugdunensis*, and received specific antibiotic treatment according to the susceptibility report (Table 4). Superinfections were detected during the follow-up with CSF cultures, and patients did not present symptoms of a new infection.

Of the total group of 17 patients with a first infection, 3 (one with *S. epidermidis*, one with *P. aeruginosa*, and one with *E. cloacae*) developed ventricular septa and were referred to the palliative care program. All eventually died (17.6%).

### 2.3. Associated Factors for Initial Healthcare-Associated Ventriculitis

The characteristics of the patients with a first infection associated with a CSF diversion procedure were compared with 20 control patients who did not develop a first infection after the surgery during the study period, and who did not have previous HAV. We found statistically significant differences related to a younger age, nutritional status (malnutrition), cause of hydrocephalus (neural tube defect), and type of ventricular drain procedure (EVD). There were no statistically significant differences in other variables, including those related to the surgical procedure (adequate timing of antibiotic prophylaxis, type of surgery (emergency or elective), number of personnel in the operating room, shift, and duration of surgery) (Table 5).

### 2.4. Outcome

Six of the seventeen patients were cured with the first antibiotic course after removal of the shunt system or EVD (35.3%); eight patients (47%) had a relapse or a superinfection and received a second course; and all were cured. A shunt system was successfully installed at the end of treatment. Three patients (one with *S. epidermidis* infection, one with *P. aeruginosa* infection, and one with *E. cloacae* infection) died in the next 90 days of their first HAV. The median total duration of antibiotic treatment, including relapses and superinfections, was 14 days (IQR: 14–19.5; minimum–maximum: 7–42 days).

## 3. Discussion

The Mexican Institute of Social Security (IMSS) provides social security to formal private sector workers and their families (approximately 50% of the population). During the COVID-19 pandemic, the network of hospitals was instructed to attend to all patients, regardless of whether they were affiliated or not with the IMSS. All non-urgent hospitalizations and elective surgeries were canceled or postponed.

The placement of a ventricular shunt is an invasive therapeutic measure necessary to manage hydrocephalus in pediatric patients, mainly in children under 12 months of age (35.3%), which carries a high risk of complications [4,9,10]. HAV is one of the most feared post-surgical complications; rates of infection vary greatly in different centers, from 1% to 45% [4,8,9,10,11,12,13]. At the beginning of and during the pandemic, HAV rates increased significantly, and the risk for patients was almost four times higher in 2021. In Mexico, there was a lack of overall personnel in the public health sector, and younger, inexperienced, and unskilled personnel were hired to deal with the pandemic [18]. In the institutions, healthcare workers with high-risk conditions (diabetes, cardiovascular or pulmonary chronic diseases, >65 years) had the option of a leave of absence. Heavy workloads, reduced staff, and longer hours were common. According to a systematic review by Koontalay A. et al., these factors can contribute to the burnout and discontinuity of healthcare practice, and can threaten patients’ safety [19]. 

This study only analyzed the first event of each patient (17 cases) who underwent a primary procedure, in order to avoid other variables that would increase the rate of infection (e.g., number of shunt system revisions, complications after a primary shunt insertion, CSF leaks, relapses). Most of the cases were registered in patients with an EVD, probably due to manipulation of the external system and poor adherence to insertion and care protocols. According to the study by Park et al. [20], the risk of infection increases with each day of catheterization. The median duration of HAV after the procedure in patients with EVD was 7 days (IQR 3–9), similar to the findings in the large cohort reported by Topjian et al., with a median time to positive culture of 7 days (IQR 4.75, 9), but a lower rate of HAV of 6% [21]. The same authors later demonstrated a significant decline in the ventriculostomy-associated CSF infection rate after the implementation of antibiotic-impregnated EVD catheters at their institution (0.9% vs. 6%, *p* = 0.00128) [22]. One study with a small number of preterm, low-birth-weight infants with post-hemorrhagic hydrocephalus found an infection rate of 7.7% (1/13) per patient and 4% (1/25) per procedure, using an antibiotic-impregnated EVD for a longer catheterization period [23].

Other authors have not reported a greater rate of EVD [13] in comparison with VPS. Ventriculo-atrial shunts and ventriculo-pleural shunts are rarely used, and the reported rates of infections are high [8]. In this study, the only patient who received a ventriculo-atrial shunt had an early infection (15 days after the procedure).

The patients, at the time of first infection, were much younger than in the control group. As is similar to other series where children under one year represent 35.3% of the cases [10], other authors found that being younger than two years old implies a 2.2 higher risk of developing ventriculitis [14]. In most of the published studies, 80% to 94% of the cases corresponded to children under 5 years [10,14,15]. All the cases in this study were ≤5 years of age. In a large 10-year study, Erps A et al. found two independent risk factors in the multivariate analysis: children who had had two or more previous revisions (odds ratio (OR) 4.8; 95% confidence interval (CI) 1.5–15.9) and those younger than 5 years of age (OR 4.5; 95% CI 1.5–13.4). Also, in the univariate analysis, a neoplastic etiology for hydrocephalus was found to be a protective factor (*p* = 0.001) [9]. In our patients with HAV, a hydrocephalus etiology due to a neural tube defect was most common (41.1%, *p* = 0.005); other etiologies had numbers too small to perform adequate comparisons.

Fever was the most frequent clinical manifestation (66.7%), followed by altered mental status. Fever is the most consistent sign in most studies, with percentages ranging from 41.5 to 90% [8,10,15]; other less frequent signs are seizures, diarrhea, vomiting, and abdominal pain. The IDSA guidelines point out that in patients with a ventriculoperitoneal shunt, fever may be the only sign of suspicion to rule out ventriculitis [8]. Regarding CSF findings, it has been described that children who have had neurosurgical manipulation may have more than five leukocytes/mm^3^ without being infected, and on the contrary, up to 20% of HAV cases may have normal values [24]. Other useful parameters for diagnosis are glucose and protein values; however, these may also be elevated in patients with hemorrhage, surgery, and trauma. For many years, the blood/cerebrospinal fluid glucose ratio has been used to diagnose meningitis and ventriculitis; a value ≤ 0.5 has a sensitivity of 80% for HAV [11,25]. In most of our cases, serum glucose levels were not available at the same moment as the CSF analysis. The CSF lactate value has also been used as a biomarker of infection in neurosurgical patients, with a sensitivity of 92% and a specificity of 88%, cutoff values between 3.4 and 5.4 mmol/L, and a positive likelihood ratio of 7.7 [26,27,28]. In this study, the lowest value was 0.06 mmol/L, but the median was 3.4 mmol/L, so it may be useful in conjunction with other parameters of CSF analysis for the diagnosis of HAV in patients with negative culture, as it is a simple, available test that is not affected by blood contamination of the CSF. Despite the fact that not all patients showed signs of infection, the blood parameters of systemic inflammation were elevated (leukocytes/mm^3^, total neutrophils and C-reactive protein). Most of the published studies do not include systemic inflammation values [9,10,29].

All the patients in the study had positive CSF cultures. Gram-positive cocci were the most commonly reported microorganisms. Coagulase-negative staphylococci were identified in 58.8% of the cases, a higher percentage than reported by other authors (23.5–33.3%) [14,15]. Also, we found a low frequency of *Staphylococcus aureus,* in contrast to that reported by Yilmas K et al. [10]. Gram-negative bacilli usually cause infections in patients with several risk factors, among them being a prolonged hospital stay, previous use of antimicrobials, frequent manipulations of the diversion system, and greater neurosurgical complexity [9,10]. In one study conducted in Indonesia with an identical number of patients, a higher frequency of Gram-negative bacilli was found (47%), but the associated factors were not studied [15]. In our hospital, the frequency of Gram-positive cocci was similar in the pre-pandemic and pandemic periods.

Multiple factors associated with the development of HAV have been described and analyzed in several studies [4,9,14,29,30]. One important contrast is that this study only included the first HAV episode, while the others included all infections. Among the main risk factors were previous revisions and manipulations of the diversion systems. One associated factor in this study, consistent with similar and larger studies, was a younger age [3,9,30]. Lee JK et al. found an HAV rate of 10.5% over a 6-year period. In the univariate analysis, a shunt performed on a patient <1 year old (relative risk (RR), 2.31; 95% CI, 1.19–4.48), and hydrocephalus due to hemorrhage (RR, 2.07; 95% CI, 1.05–4.06) demonstrated statistical significance, but in the multivariate analysis, only an age <1 year (RR, 2.23; 95% CI, 1.06–4.69) was an independent risk factor [30]. The etiology of hydrocephalus varies according to the care center. In our hospital, patients with neural tube defects accounted for more than 40% (*p* = 0.005). Erps A et al. [9] found that a neoplastic etiology for hydrocephalus was a protective factor (*p* = 0.001).

This study did not find statistically significant differences in the operative variables—shift (morning, evening, or night), type of surgery (emergency or elective), adequate timing of antibiotic prophylaxis (60 min before skin incision), number of personnel in the operating room (median of 7 for both groups), and duration of surgery—between the HAV patients and the control patients. A low compliance with the timing requirements for antibiotic prophylaxis was evident. In a systematic review and meta-analysis to evaluate the effectiveness of antibiotic prophylaxis in children who underwent placement of intracranial ventricular shunts, the authors included 7 studies with 694 participants; compared with the control group, antibiotic prophylaxis showed a significant difference in terms of the reduction in infection rate (RR = 0.59, 95 % CI = 0.38, 0.90, *p* < 0.05) [31]. An intervention review conducted by Cochrane to determine the effect of different routes of antibiotic prophylaxis (i.e., oral, intravenous, intrathecal, topical, and via antibiotic impregnated shunt catheters) on CSF shunt infections in patients with internalized CSF shunts included a total of 11 small randomized controlled trials (three in children), which contained 1109 participants. Only intravenous administration of antibiotic prophylaxis seemed to have an effect on the risk of shunt infections (RR 0.55, 95% CI 0.33 to 0.90), but the quality of the included studies was low. The authors concluded that it is uncertain whether the prevention of shunt infection varies according to different antibiotic agents, different administration routes, timing, and doses, or by the characteristics of the patients (children and adults) [32]. Currently, most of the guidelines for the prevention of SSI recommended the use of prophylaxis in neurosurgery. In Mexico, cefazolin is not available, so cephalothin is used as the first choice.

The outcome for patients with HAV was poor. Only 35.3% were cured with the first antibiotic course. The diversion devices were removed, and the patients received systemic targeted therapy according to the susceptibility patterns of the isolated bacteria and the recommendations of the clinical guidelines. There have been no prospective, randomized controlled trials to evaluate a superior treatment or optimal administration route for shunt infections. In a 10-year study in Turkey, 38 patients received systemic plus intraventricular (IVT) antibiotics (group A), and 40 patients only received systemic treatment (group B). The authors found that IVT accelerated clinical and microbiological recovery and shortened the duration of CSF sterilization and hospital stay compared to patients receiving only systemic antibiotics. But intraventricular treatment was added to IV treatment after an average of 5.7 days (between 3 and 16 days) due to the absence of improvement in CSF parameters, positive cultures, and clinical failure [33]. Thus, the real effectiveness of intraventricular antibiotics could not be evaluated, as group A and B were not comparable. In previous decades, intraventricular injection of antibiotics was related to significant toxicity. In light of infections due to multi-resistant gram-negative bacteria, several publications aimed to review its role [34,35,36]. Still, the recommendations are not supported by controlled clinical trials. Only one patient in this study received intraventricular amikacin due to the persistence of positive CSF cultures.

Few studies have reported the outcomes at the end of treatment [10,30,33]; in most of the patients, a VPS was installed or re-installed. Besides the elevated costs, length of hospital stay, and risk of superinfections, the mortality rates can be as high as 20.4% in Turkey [10], which is similar to the result of the present study (17.6%). This rate is lower in Argentina (9%) [37] and nonexistent in Chile [13]. When comparing risk factors associated with adverse outcomes in adult and pediatric patients with HAV and meningitis, children have better outcomes. In one of the largest studies, good recovery was observed in 30 of 49 (61.2%) children and 18 of 166 (10.8%) adults, and having a ventriculoperitoneal (VP) shunt-associated infection (OR, 0.17; 95% CI, 0.08–0.33; *p* < 0.001) was associated with a lower risk of adverse clinical outcomes. The overall mortality rate was 9.3%, but the study did not specify deaths according to group [38].

Mexico reported the first case of COVID-19 on 27 February 2020. It is ranked in 18th place worldwide, with 7,633,355 cumulative cases and 334,336 deaths. Only 44.2% of the population has received at least one booster or additional dose of the vaccine [39]. Surgical patients suffered the consequences of the disrupted care services. Adult and pediatric neurosurgical procedures showed a decrease in operative volume, a reduction in neurosurgical activity, and an increased rate of postoperative infections [40,41,42,43]. In an adult reference center in Mexico City, the rate of infections increased from 3.5% in 2019 to 5.6% in 2020 (*p* = 0.002). Of particular interest were the reported CSF shunt infections (SSI meningitis) and HAV, representing 29.7% (36/121) of all infections during 2020–2022, with a rate of 5.9% for the CSF diversion surgeries [41].

Major changes need to be implemented to prevent HAV. For example, in a level 1 trauma center, an EVD care bundle has been implemented to reduce infection rate. Some of the practices include strict asepsis with full barrier precautions for procedures involving EVD manipulation; minimizing EVD handling; stopping routine culturing; using standardized EVD dressings and changing dressings only when compromised; and development of an EVD transport protocol. After the implementation of the bundle, the rate of EVD-associated ventriculitis decreased from 8.8 per reported EVD days in 2019 to 0 per reported EVD days in 2021 [44]. A combination protocol and different strategies need to be evaluated after implementation in order to achieve reduction in infection rates, which can take years [45].

This study has several limitations. The number of HAV infections and controls was limited, but most of the patients in previously published studies had a history of at least one previous infection. Also, we did not find additional controls that fulfilled the criteria of a primary diversion procedure without HAV. A strength of the study is that all cases had a confirmed diagnosis of HAV with a positive CSF culture. Detailed information on HAV and the outcomes in pediatric patients is limited. These findings may contribute to previously published data and highlight the importance of preventive measures in pediatric patients from the first CSF diversion procedure.

## 4. Materials and Methods

A retrospective cross-sectional study was performed after receiving the approval of the Institutional Review Board. The study period lasted from January 2021 to December 2022, and it was conducted at the Pediatric Hospital, National Medical Center, XXI century, a tertiary-care-level reference pediatric hospital. During the COVID-19 pandemic, the hospital converted to a hybrid facility and provided care for COVID-19 patients and a limited number of high-specialty patients. Before the conversion, the hospital had 184 beds and 2 intensive care units (neonatal and pediatric). During the emergency, a special in-patient unit with 6 beds and an area with 40 beds were allowed to receive patients with SARS-CoV-2 infections. Any pediatric patient classified as a suspected case of COVID-19, regardless of their health insurance status, was evaluated at the emergency department. A screening protocol was established for all patients before admission. After the review of the available literature, a management consensus by the infectious disease specialists of the hospital was published in April 2020 [46].

The rates of HAV in patients with CSF diversion procedures (EVD, VPS, or ventriculo-atrial shunt) performed in the hospital before (2017–2019), at the beginning of (2020), and during the pandemic (2021–2022) were registered and compared.

The records of all patients who underwent a primary CSF diversion procedure for the treatment of hydrocephalus from January 2021 to December 2023 were reviewed. The inclusion criteria were patients aged 0–17 years who had developed HAV up to 30 days after EVD and up to one year after VPS or ventriculo-atrial shunt, and these patients formed the study group (cases). We included patients with either congenital or acquired hydrocephalus. The control group was formed of patients who did not present with ventriculitis, with a primary CSF diversion procedure performed during the same time period. A primary CSF diversion procedure was defined as an initial surgery with no past history of shunt revisions.

The definition of healthcare-associated ventriculitis according to the Centers for Disease Control and Prevention’s National Healthcare Safety network (CDC/NHSN) and IDSA guidelines was used [8,47], according to which at least one of the following criteria must be met: (a) an organism cultured from CSF; (b) at least two of the following symptoms with no other recognized cause in patients aged >1 year: fever > 38 °C or headache, meningeal signs, or cranial nerve signs, or at least two of the following symptoms with no other recognized cause in patients aged ≤1 year: fever > 38 °C or hypothermia < 36 °C, apnea, bradycardia, or irritability and (c) is at least one of the following: increased white cells; elevated protein; decreased glucose in CSF; organisms seen on a Gram stain of the CSF; and organisms cultured from blood or a positive nonculture diagnostic laboratory test from the CSF, blood, or urine.

Demographic data, intraoperative variables, clinical characteristics of infection, antibiotic treatment, and outcome information were extracted from the medical records. Cephalothin was the standard antibiotic recommended for perioperative prophylaxis. According to the hospital guidelines, the first dose should be administered within 60 min before the skin incision, and two more doses should be given in the next 24 h to be considered as adequate.

A comparison of the variables of patients with ventriculitis and patients without ventriculitis was performed to identify factors associated with the development of infection. Superinfection was defined as a new shunt infection with a different pathogen during the therapy for the first infection. Relapse was defined as reinfection with the same pathogen after two negative CSF cultures and before the reimplantation of a new CSF shunt.

No sample size calculation was performed; all patients who met the case or control criteria during the study period were included, by non-probability consecutive sampling.

### Statistical Analysis

Descriptive statistics with frequencies and percentages were used. Medians and interquartile ranges (IQR) were used for variables without normal distribution, and the normality of distribution was determined using the Shapiro–Wilk test. We performed univariate analysis with the Chi-square test, the Chi-square test with Bonferroni post hoc adjustment Chi-square for trends; Fisher’s exact test to compare proportions for categorical variables; and the Mann–Whitney U test to compare medians for continuous variables. A *p* value of ≤0.05 was considered statistically significant. All analyses were conducted using StataCorp. 2021. Stata Statistical Software: Release 17 (StataCorp LLC: College Station, TX, USA).

## 5. Conclusions and Future Perspectives

The rate of HAV was high in the two years after the beginning of the pandemic. Most cases presented in children <1 year with an EVD. None of the intraoperative factors showed statistically significant differences. Only one-third of the patients were cured with the first antibiotic course. Relapses and superinfections were common complications. The use of standardized protocols for infection prevention is indispensable, most of all for EVD insertion. A careful consideration of the use of intraventricular antibiotic treatment needs to be addressed, as therapeutic success is low with the first course of systemic antibiotics.

## Figures and Tables

**Table 1:** Total number of healthcare-associated ventriculitis and CSF diversion procedures in 2017–2022.

Year	Cases of HAV (% Rate of Infection)	Total Number of CSF Diversion Procedures	Odds Ratio *
2017	9 (8.6)	108	1.0 (reference value)
2018	9 (11.5)	78	1.43
2019	13 (14)	94	1.76
2020	15 (20.2)	74	2.79
2021	25 (26.5))	94	3.98
2022	22 (20.9)	105	2.91

* Chi-square for linear trend *p* value = 0.00033; HAV = healthcare-associated ventriculitis; CSF = cerebrospinal fluid.

**Table 2:** Clinical characteristics and outcomes of pediatric patients with a first incidence of healthcare-associated ventriculitis.

Variable	Total N = 17
	n (%)
Male	13 (76.5)
Age (months) median (IQR)	9 (1, 60)
Malnutrition	4 (23.5)
**Cause of hydrocephalus**	
-Neural tube defect	7 (41.1)
-Other congenital causes	4 (23.5)
-Brain tumor	4 (23.5)
-Intraventricular hemorrhage	2 (11.7)
**CSF drain procedure**	
-External ventricular drain	10 (58.8)
-Ventriculo-peritoneal shunt	6 (35.3)
-Ventriculo-atrial shunt	1 (5.9)
Surgical prophylaxis (adequate timing)	5 (29.4)
**Prophylactic antibiotics**	
-Cephalothin	12 (70.5)
-Cefotaxime	2 (11.7)
-Vancomycin	2 (11.7)
-Ampicillin	1 (5.8)
Duration of surgery (min) median (IQR)	63 (60, 131)
Patients with signs or symptoms *	11 (68.8)
-Fever	8 (66.7)
-Altered mental status	3 (25)
-Seizures	2 (16.7)
-Emesis	2 (16.7)
-Diarrhea	1 (8.3)
**Bacteria in CSF culture**	
**-Gram-positive cocci**	12 (70.6)
-*Staphylococcus epidermidis*	9 (75)
-*Staphylococcus aureus*	2 (16.7)
-*Staphylococcus saprophyticus*	1 (8.3)
**-Gram-negative bacilli**	5 (29.4)
-*Escherichia coli*	3 (60.0)
-*Pseudomonas aeruginosa*	1 (20.0)
-*Enterobacter cloacae*	1 (20.0)
**Outcome**	
-Cured	6 (35.3)
-Relapse	4 (23.5)
-Superinfection	4 (23.5)
-Palliative care	3 (17.6)

IQR = interquartile range. CSF = cerebrospinal fluid. * Some patients presented with more than one sign or symptom.

**Table 3:** Cerebrospinal fluid and blood laboratory findings in patients with healthcare-associated ventriculitis.

Parameter	Median	Minimum–Maximum	Interquartile Range
**CSF**			
Leukocytes/mm^3^	150	3–11,194	36.25–562
PMNs, %	83	8.3–90.3	24.2–83.8
Glucose, mg/dL	56	0.1–73.2	30–59.5
Protein, mg/dL	93.5	4.5–287	57.8–410.5
Lactate, mmol/L	3.4	0.06–10.8	1.99–5.63
**Blood**			
White blood cell count/mm^3^	15,175	5420–33,000	10,150–20,547
Neutrophils/mm^3^	8295	2040–24,380	4342–17,700
Lymphocytes/mm^3^	3535	670–8870	2005–6407
C-reactive protein, mg/L	63	0.59–442	7.7–250
Glucose, mg/dL	101.5	45.6–189	89.75–136

CSF = cerebrospinal fluid. PMNs = polymorphonuclear neutrophils.

**Table 4:** Characteristics of patients with relapse or superinfection.

Variable	N = 8
	n (%)
Male Sex	6 (75)
Age (months) median (IQR)	7 (2, 15)
Cause of hydrocephalus	
-Neural tube defect	5 (62.5)
-Intraventricular hemorrhage	2 (25)
-Brain tumor	1 (12.5)
Initial diversion procedure	
-External ventricular drain (EVD)	4 (50)
-Ventriculo-peritoneal shunt	4 (50)
Relapse	
*-S. epidermidis*	2 (25)
*-S. aureus*	1 (12.5)
*-E. cloacae*	1 (12.5)
Superinfections	4 (50)
Cured	8 (100)

IQR: interquartile range; CNS: central nervous system; CSF: cerebrospinal fluid.

**Table 5:** Associated factors in patients with and without healthcare-associated ventriculitis.

Variable	Ventriculitis Patients (n = 17)	Control Patients (n = 20)	Total (N = 37)	*p* ^1^
	**n (%)**			
Male sex	13 (76.5)	12 (60.0)	25 (67.6)	0.31
Age (months) median (IQR)	9 (1, 60)	102 (30, 144)	60 (9, 132)	<0.01 ^2^
**Nutritional status**				
-Normal	13 (76.5)	20 (100)	33 (89.1)	0.03 ^3^
-Malnutrition	4 (23.5)	0	4 (10.8)	
**Hydrocephalus etiology**				
-Neural tube defect	7 (41.1)	1 (20)	8 (21.6)	0.015
-Other causes *	4 (23.5)	8 (10)	12 (10.8)	
-Brain tumor	4 (23.5)	10 (50)	14 (37.8)	
-Intraventricular hemorrhage	2 (11.8)	1 (20)	3 (8.1)	
**CSF drain procedure**				
-External ventricular drain (EVD)	10 (58.8)	2 (10)	12 (32.4)	0.03
-Ventriculo-peritoneal shunt	6 (35.3)	18 (90)	24 (64.9)	
-Ventriculo-atrial shunt	1 (5.9)	0	1 (2.7)	-
Surgery performed during morning shift	11 (64.7)	10 (50)	21 (56.7)	0.28
Evening shift	3 (17.6)	5 (25)	8 (21.6)	
Night shift	3 (17.6)	5 (25)	8 (21.6)	
Emergency surgery	11 (64.7)	14 (70)	25 (67.5)	0.71
Adequate surgical prophylaxis (timing)	5 (29.4)	9 (45)	14 (37.8)	0.49 ^3^
Duration of surgery (min) median (IQR)	63 (60, 131)	98 (71, 144)	85 (63, 143)	0.28 ^2^
Personnel in operating room median (IQR)	7 (6, 7)	7 (6, 7)	7 (6, 7)	0.45 ^2^
Median surgical bleeding in mL (IQR)	5 (5, 15)	13 (5, 35)	5 (5, 20)	0.05 ^2^

^1^ Chi-square test or Bonferroni-corrected *p*-values; ^2^ Mann–Whitney U test; ^3^ Fisher’s exact test; IQR: interquartile range. * Other causes include vascular malformations, congenital aqueduct stenosis, developmental cysts, posterior fossa malformations, and subarachnoid hemorrhage.

## Data Availability

The data presented in this study are available upon request from the corresponding author.
